# Analysis of Factors Associated with the Postoperative Healing of Medication-Related Osteonecrosis of the Jaw in Patients with Osteoporosis

**DOI:** 10.3390/jcm10163651

**Published:** 2021-08-18

**Authors:** Young-Ho Shim, Sang-Hwan Jung, Obida Boboeva, Sung-Tak Lee, Jin-Wook Kim, Tae-Geon Kwon, So-Young Choi

**Affiliations:** Department of Oral and Maxillofacial Surgery, School of Dentistry, Kyungpook National University, 2177 Dalgubeol-daero, Jung-gu, Daegu 41940, Korea; asimjjang@naver.com (Y.-H.S.); hello909@naver.com (S.-H.J.); boboyevaobida@gmail.com (O.B.); st0907@knu.ac.kr (S.-T.L.); vocaleo@knu.ac.kr (J.-W.K.); kwondk@knu.ac.kr (T.-G.K.)

**Keywords:** MRONJ, surgical treatment, postoperative healing, sequestrectomy, primary closure

## Abstract

Background: Surgical treatment is considered the best approach by many researchers for medication-related osteonecrosis of the jaws (MRONJ). While postoperative outcomes are mainly favorable, wound healing still fails in some cases. This retrospective study aimed to evaluate the factors affecting the postoperative healing of MRONJ. Methods: This study involved 400 osteoporosis patients who received surgical treatment from January 2009 to January 2018 in Kyungpook National University Hospital. The patient, drug, and clinical factors were collected as investigation variables. The obtained data were statistically analyzed to identify relationships between the factors and healing aspect. Results: Univariate logistic regression analysis showed that the route of drug administration, bone exposure, types of surgical management, and wound management had a significant influence (*p* < 0.05) on the healing outcome. Sequestrectomy with primary closure had a more positive effect on favorable healing. In the multivariate logistic regression test, the effect of wound management alone was not statistically significant (*p* > 0.05). Conclusion: In patients with osteoporosis, the factors such as intravenously administered drugs, fistulas that were probed to the bone, and surgical management with curettage were associated with a lower rate of postoperative complete healing of MRONJ, whereas primary closure of wounds led, possibly, to good healing outcomes. The strengths of the study include its relatively large sample size and that its results can hopefully aid in the clinical decisions for practitioners and future research studies for researchers.

## 1. Introduction

It has not been long since the relationship between osteonecrosis of the jaws (ONJ) and antiresorptive drugs such as denosumab and bisphosphonates was first described [1,2,3]. While these drugs are used to treat bone metastases and osteoporosis, these have been affecting the quality of life related to oral health, Refs. [1,4,5,6] making daily dental treatment more challenging.

However, it should be noted that other medications such as tocilizumab, tyrosine kinase inhibitors, monoclonal antibodies, mammalian target of rapamycin inhibitors, radiopharmaceuticals, selective estrogen receptor modulators, and immunosuppressants have, also, been mentioned as a potential cause of ONJ in the literature [7].

Although the pathophysiological characteristics of ONJ have been hypothesized, the exact mechanism of how this disease develops remains unclear. According to one study, the interaction of several factors such as infection and inflammation, lack of immune resilience, soft tissue toxicity, alterations in angiogenesis, and disturbances in bone remodeling may lead to the development of ONJ [8]. 

A generally accepted treatment strategy for MRONJ has not yet been established [1,9,10,11] because of challenges in the accurate staging of the disease [12]. Schiodt et al. also suggested using the term “non-surgical treatment” instead of “conservative treatment” [12]. The therapeutic goal in MRONJ is complete mucosal coverage, regardless of the choice between non-surgical or surgical treatment. This is to eliminate secondary infection through the exposed bone and to have perfect saliva proof. Many studies have been done on how treatment strategies achieve this therapeutic goal. However, it is still controversial whether a non-surgical or surgical treatment has a better prognosis [11]. 

Interestingly, recent literature suggests that surgical treatment may be a better treatment option to promote mucosal healing and long-term outcomes [4,12]. Therefore, interest in treatment outcome of surgically managed MRONJ has been increased in recent years. The influence of several factors on the treatment outcome was studied by Hayashida et al. [13] in 361 patients while Ruggiero and Kohn [2] investigated 337 patients. However, most studies have limitations such as small sample size [10,14,15,16].

This study aimed to investigate the effect of several factors on the postoperative healing of a relatively large number of MRONJ stage II and III patients with osteoporosis.

## 2. Materials and Methods

### 2.1. Subjects

We initially selected 544 patients who were pathologically diagnosed with MRONJ at Kyungpook National University Hospital, from January 2009 to January 2018. Then, 88 patients were excluded because did not undergo surgery after diagnosis. Patients were excluded if they had undergone treatment other than surgical curettage and sequestrectomy, such as incision drainage and biopsy alone. Recurrence surgery cases were also excluded, as well as 20 subjects who could not remember exact the time period of taking bisphosphonates. Finally, the subjects included for analysis were biopsy-proven MRONJ patients who had taken medication for at least 6 months intravenously (IV) or 12 months orally and underwent surgery. MRONJ cases were either stage II or III [17]. The follow-up period was at least 3 months after surgery. 

Of the remaining 432 patients, 30 received antiresorptive drugs for cancer treatment and 2 patients with other non-malignant diseases were excluded so that the results would be more specific. A total of 400 subjects with osteoporosis were thus included in the analysis. This research was approved by the Institutional Review Board of Kyungpook National University Dental Hospital (reg. no. KNUDH-2021-03-01-00).

### 2.2. Variables

Several clinical factors (age, sex, route and type of drug administered, MRONJ stage, bone exposure: Probes to bone or bone exposed [17], type of surgical management, wound management, and healing) were retrieved from the patients’ medical records as variables for the analysis. There are some cases wherein the sequestrum was not observed on panoramic standard or cone-beam computed tomography, but a visible sequestrum was in the lesion intraoperatively. Such cases were classified as having no sequestrum on radiologic findings, but sequestrectomy was listed as the surgical treatment method.

### 2.3. Treatment Methods

All patients underwent surgical treatment with conservative treatment such as education to improve oral hygiene, if necessary, and antibiotic mouth rinse or antibiotic medication. Empirical antibiotics were used when infection was evident at the first visit. In patients with pus discharge, appropriate antibiotics were administered according to the results of pus culture. Surgery was done under local or general anesthesia. Surgical management consisted of two techniques: sequestrectomy and surgical curettage. If sequestrum was confirmed on cone-beam computed tomography, sequestrectomy was performed; in cases in which no sequestrum was found and osteolytic changes were evident, performing curettage was planned. Sequestrectomy involved reflecting a flap to expose the entire affected area, followed by removal of all sequestrum until a fresh, unaffected bony margin is obtained. In surgical curettage, the affected area was curetted well using a surgical curette until all the necrotic bone and inflammatory tissue were removed and the bone margins were rounded off. Wound management was done via primary closure or secondary healing with an open wound. 

The response of the treatment was evaluated by classifying all the cases into three categories:

1. Complete healing: complete regrowth of the oral mucosa over the previously exposed bone for at least 3 months with no suppuration present.
2. Partial healing: only a pinpoint exposed bone was observed for at least 3 months and no suppuration was present.
3. No improvement/no response: no improvement in the clinical signs, with no effect of the surgical management after 3 months of surgery.

### 2.4. Statistical Analysis

Descriptive statistics are presented in means, standard deviation, and frequencies. Spearman correlation analysis was used to identify relationships between the aforementioned factors and healing. The factors with significant correlations were entered into the univariate and then multivariate ordinal logistic regression model under the Generalized Linear Models to distinguish the effect of the factors on healing aspects of MRONJ surgical treatment. Statistical analysis was performed using the standard software (SPSS, version 25, IBM Corp., Armonk, NY, USA). The significance level was set at *p* < 0.05.

## 3. Results

**Figure 1:**
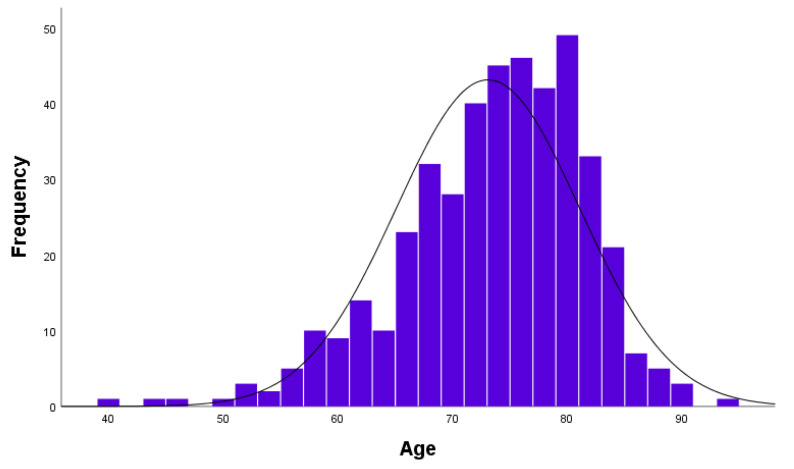
Age distribution in histogram.

**Figure 2:**
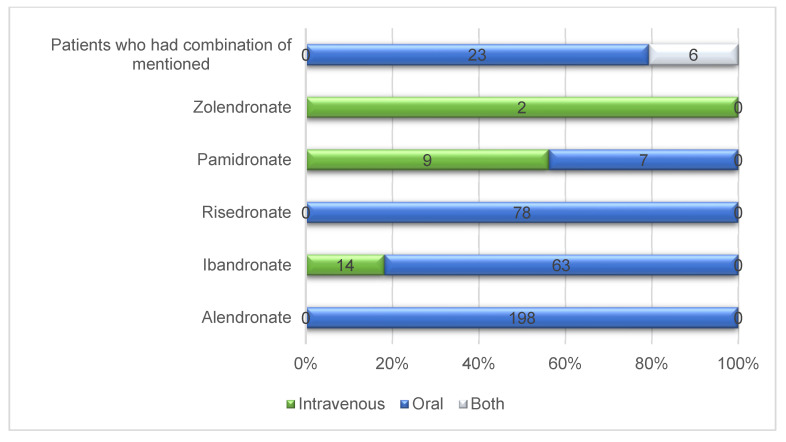
Distribution of bisphosphonates by administration route.

**Figure 3:**
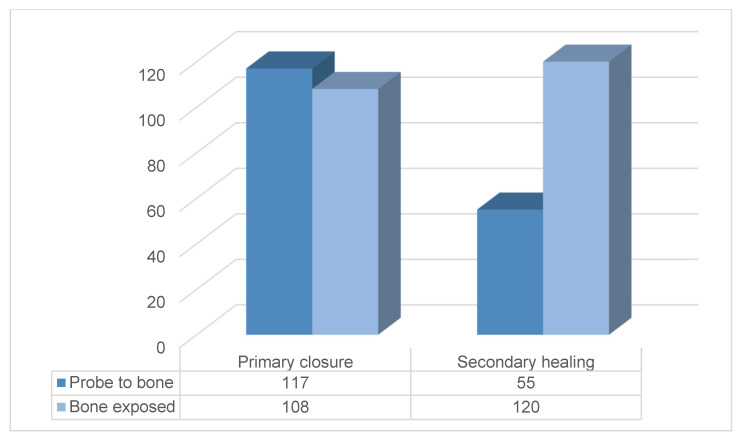
Bone-exposed patients distributed by wound management.

The demographic and clinical characteristics of the study subjects are shown in Table 1. Their ages ranged from 40 to 93 years, with a mean of 73.89 ± 7.31 years (Figure 1). There was prevalence of female patients (95.5%) and a mandibular location of the MRONJ (68.5%). Among the bisphosphonates, alendronate was the most common drug consumed by the study subjects (49.5%). The duration of drug administration among patients ranged between 6 and 480 months with a mean of 58.16 ± 54.70 months, and 369 patients (92.3%) took these bisphosphonates orally. Pamidronate was the most commonly administered IV drug (Figure 2). In 332 patients (83.0%), the formation of sequestrum was confirmed by radiologic examination at the first visit. However, sequestrectomy was performed in 340 patients (85.0%). This is because, among those described to have no visible sequestrum at first visit (n = 68), eight patients had a detectable sequestrum on preoperative examination. After three months postoperatively, 316 patients (79.0%) were completely healed, 76 were partially healed, and only eight had no improvement. Figure 3 shows the distribution of patients with bone exposed and probes to bone according to wound management.

Spearman correlation analysis showed that the route of drug administration, bone exposure, types of surgical management, and wound management were all significantly correlated (*p* < 0.05) with healing (Table 2).

The effects of each of these variables were analyzed using the univariate ordinal logistic regression analysis; the odds ratios (OR) are listed in Table 3. The probability of complete healing was less in the group of patients who had IV-administered drugs (0.374 times compared to oral), with bone exposed (0.398 times compared to probes to bone), and who had undergone surgical curettage (0.451 times compared to sequestrectomy). Primary closure of the surgical wound had a more favorable healing effect than open-type wound management (OR: 1.620, 95% CI: 1.000–2.624).

In the multivariate model of ordinal logistic regression analysis, the effect of wound management alone was not statistically significant (*p* > 0.05) (Table 4).

## 4. Discussion

MRONJ is a recently discovered disease, and its exact etiopathogenesis has not been clarified. Therefore, questioning its causes and pathophysiological characteristics has become a focus of research. Multiple factors may play a considerable role in the development of MRONJ [8,18]. Clinically, the type and dose of antiresorptive drugs and the duration of administration have been considered as risk factors [18]. An in vitro study showed impairment of osteogenic differentiation of stem cells from the human periodontal ligament when zoledronic acid is infused in high dose and for a long duration [19]. However, in another in vitro study of mesenchymal stem cells from osteoporotic patients, the researchers investigated alendronate, ibandronate, and zoledronate, and results suggest the importance of patient-specific responses rather than drug type in producing favorable osteogenic differentiation [20].

Nevertheless, the risk factor for the development of MRONJ that has been most often considered is infection. A recent systematic review demonstrated that infection was a major risk factor because diagnostic signs and prevention and treatment measures were all focused on infection [18]. In addition to avoiding infection and other therapeutic-prophylactic measurements to reduce MRONJ occurrence, a drug holiday before surgery was highly emphasized in an animal study [21].

The current literature suggests various treatment strategies for MRONJ depending on its stage and other clinical parameters of the patient [3,6,11,22]. Despite non-surgical treatment options, the surgical approach is still considered the best treatment choice of MRONJ [10,12,13,23,24,25]. However, unfavorable outcomes are inevitable even with the surgical approach. To our knowledge, there are only a few published studies investigating factors impacting treatment outcomes in a considerably large sample size [2,13,14,25,26]. Therefore, we aimed to examine an association between multiple variables with postoperative healing in a relatively large sample size of surgically treated MRONJ patients with osteoporosis.

Both univariate and multivariate regression analysis showed a significant association between routes of drug administration with healing outcomes. Although a large proportion of patients were exposed to oral bisphosphonates, patients who received IV-administered drugs were 0.316 times less likely to have favorable healing outcomes in the multivariate analysis. This was inconsistent with the findings of previous reports [2,14,24]. This can be associated with the amount of drug received by the bone cells and interactions with osteoclasts. Oral bisphosphonates are poorly absorbed through the digestive tract, and thus there is less dose in the plasma compared to IV administration [2]. This can be the possible reason why both univariate and multivariate regression analyses showed that this factor had an insignificant effect on healing outcomes in patients with osteoporosis, most of whom were taking oral antiresorptive drugs.

Studies have conflicting results regarding the association between the MRONJ stage and treatment outcomes. While some [2,24] found a significant effect of the disease stage on treatment outcomes, a majority [13,14,25,26] claim that these are unrelated. In agreement with the latter, this study found no significant relationship between the stage of MRONJ and healing. However, patients who had MRONJ with exposed bone had significantly less positive outcomes than those with fistulas that probed to the bone. This can be related to the effect of wound management, since the majority of patients with bone exposure were managed with secondary healing of the wound, which was to have less favorable healing outcomes on the univariate regression analysis. Similarly, Kang et al. [16] found a significantly better success rate in MRONJ patients managed with primary closure compared to secondary healing. In contrast, wound management was not significantly related to treatment outcomes in a study conducted by Hayashida et al. [13]. In the presence of bone exposure, primary closure is often difficult due to a lack of soft tissue volume. Stage III cases especially have a greater amount and severity of infected soft tissue than stage II. Thus, primary closure can be relatively difficult. In stage II, the infected soft tissue is relatively small, and so primary closure can achieve complete closure of the surgical wound. Therefore, primary closure can be expected to have better postoperative outcomes and should be preferred in MRONJ cases, especially in stage II cases and those with exposed bone.

The surgical treatment modalities of MRONJ range from superficial debridement accompanied by antibiotic therapy to extensive bone resection [27,28]. The subjects in our study cohort had undergone either sequestrectomy or curettage for the treatment of MRONJ. After comparing their outcomes, surgical curettage had a less positive effect on healing than sequestrectomy. This was in line with the findings of a previous study [26]. Complete removal of any necrotic bone is a major step in treatment because otherwise, the risk of disease recurrence or progression would remain [29,30,31]. When surgical treatment is performed in MRONJ patients, the extent of bone resorption is always questionable [30]. However, better treatment outcomes were reportedly obtained in cases undergoing minimally invasive surgical procedures [32]. Fluorescence-guided surgery has been suggested to be effective in detecting the extent of necrotic bone during the surgery of MRONJ. Nevertheless, the effect of this method on mucosal healing was not better than that of conventional surgical methods [33]. Furthermore, according to a recent systematic review, application of the platelet concentrates not only helps prevent MRONJ but also enables better healing outcomes [34].

This study is the first to gather data related to this topic with a relatively large sample size compared to previous studies. However, regarding limitations, the retrospective character of this study may have introduced bias during data collection. Furthermore, this study would have been more comprehensive if it analyzed more factors such as systemic diseases, initiating events as etiology (trauma, dental treatments, or spontaneous), and size of the defect. Therefore, further investigations with a broader scope are needed to have a better grasp of the topic at hand.

## 5. Conclusions

Both clinicians and researchers need to know the reasons for unfavorable postoperative healing of MRONJ in some cases. This study showed that the route of drug administration (IV, oral, or both), the degree of bone exposure (probes to bone or exposed), types of surgical management (sequestrectomy or curettage), and wound management (primary closure or secondary healing) all have significant effects on the postoperative healing of surgically treated MRONJ in osteoporotic patients. Intravenously administered drugs, probing of fistulas to the bone, and surgical management with curettage were associated with a lower rate of postoperative healing of MRONJ, whereas primary closure of wounds led to good healing outcomes in patients with osteoporosis. The strengths of the study include its relatively large sample size and that its results can aid in clinical decisions made by practitioners and future research studies.

## Figures and Tables

**Table 1:** Descriptive characteristics of the study variables.

Factors	Total (%)
Age	
Mean ± SD	73.89 ± 7.31
Median (IQR)	75.00 (9)
Sex	
Male	18 (4.5)
Female	382 (95.5)
Site	
Maxilla	107 (26.8)
Mandible	274 (68.5)
Both jaw	19 (4.8)
Bisphosphonates	
Alendronate	198 (49.5)
Ibandronate	77 (19.3)
Risedronate	78 (19.5)
Pamidronate	16 (4.0)
Zoledronate	2 (0.5)
Patients who had combination of abovementioned	29 (7.2)
Route of administration	
Intravenous	25 (6.3)
Oral	369 (92.3)
Both	6 (1.5)
Duration of administration (month)	
Total	
Mean ± SD	58.16 ± 54.70
Median (IQR)	40.50 (48)
For intravenous administration	
Mean ± SD	31.24 ± 27.53
Median (IQR)	24.00 (25)
For oral administration	
Mean ± SD	59.58 ± 55.83
Median (IQR)	48.00 (48)
For both administration	
Mean ± SD	82.50 ± 34.77
Median (IQR)	81.00 (49)
MRONJ stage	
II	228 (57.0)
III	172 (43.0)
MRONJ Bone exposure	
Probes to bone	172 (43.0)
Bone exposed	228 (57.0)
Sequestrum formation	
Formed	332 (83.0)
None	68 (17.0)
Types of surgical management	
Sequestrectomy	340 (85.0)
Surgical curettage	60 (15.0)
Wound management	
Primary closure	225 (56.3)
Secondary healing	175 (43.7)
Healing aspect	
Complete healing	316 (79.0)
Partial healing	76 (19.0)
No improvement	8 (2.0)

SD—standard deviation; IQR—interquartile range.

**Table 2:** Description of treatment outcomes and Spearman’s rho correlation analysis of the factors and healing.

Factors	Healing			*p*-Value
	Complete Healing	Partial Healing	No Improvement	
Age (mean, SD)	73.86 ± 7.24	74.18 ± 7.62	72.25 ± 7.89	0.680
Sex				0.621
Male	15	3	0	
Female	301	73	8	
Site				0.287
Maxilla	88	17	2	
Mandible	214	55	5	
Both jaw	14	4	1	
Bisphosphonates				0.382
Alendronate	160	35	3	
Ibandronate	59	16	2	
Risedronate	62	14	2	
Pamidronate	10	6	0	
Zoledronate	0	2	0	
Patients who had combination of abovementioned	25	3	1	
Route of administration				0.030 *
Intravenous	15	9	1	
Per oral	296	67	6	
Both	5	0	1	
Duration of drug administration	56.77 ± 52.83	55.13 ± 56.56	54.11 ± 35.90	0.870
MRONJ stage				0.284
Stage II	176	46	6	
Stage III	140	30	2	
MRONJ Bone exposure				0.001 *
Probes to bone	150	19	3	
Bone exposed	166	57	5	
Types of surgical management				0.006 *
Sequestrectomy	277	55	8	
Surgical curettage	39	21	0	
Wound management				0.049 *
Primary closure	186	34	5	
Secondary healing	130	42	3	

* *p* < 0.05.

**Table 3:** Univariate ordinal logistic regression analysis.

Factors	Odds Ratio	95% CI		*p*-Value
		Lower	Upper	
Route of administration (ref.: per oral)				
Intravenous	0.374	0.163	0.856	0.020 *
Both way	1.007	0.112	9.058	0.995
MRONJ Bone exposure (ref.: Probes to bone)				
Bone exposed	0.398	0.234	0.679	0.001 *
Types of surgical management (ref.: sequestrectomy)				
Surgical curettage	0.451	0.250	0.812	0.008 *
Wound management (ref.: secondary healing)				
Primary closure	1.620	1.000	2.624	0.050 *

CI–confidence interval, * *p* < 0.05.

**Table 4:** Multivariate ordinal logistic regression analysis.

Factors	Odds Ratio	95% CI		*p*-Value
		Lower	Upper	
Route of administration (ref.: per oral)				
Intravenous	0.316	0.134	0.745	0.008 *
Both way	0.853	0.092	7.940	0.889
MRONJ Bone exposure (ref.: Probes to bone)				
Bone exposed	0.404	0.231	0.705	0.001 *
Types of surgical management (ref.: sequestrectomy)				
Surgical curettage	0.443	0.242	0.811	0.008 *
Wound management (ref.: Secondary healing)				
Primary closure	1.395	0.840	2.315	0.199 *

CI–confidence interval, * *p* < 0.05.

## Data Availability

The data presented in this study are available in the article.
